# Integrated Analysis of Mismatch Repair System in Malignant Astrocytomas

**DOI:** 10.1371/journal.pone.0076401

**Published:** 2013-09-20

**Authors:** Irene Rodríguez-Hernández, Juan Luis Garcia, Angel Santos-Briz, Aurelio Hernández-Laín, Jose María González-Valero, Juan Antonio Gómez-Moreta, Oscar Toldos-González, Juan Jesús Cruz, Javier Martin-Vallejo, Rogelio González-Sarmiento

**Affiliations:** 1 Molecular Medicine Unit, Department of Medicine, University of Salamanca, Salamanca, Spain; 2 IBMCC and IBSAL, (USAL/CSIC/University Hospital), Salamanca, Spain; 3 Institute for Health Science Studies of Castilla y León, Salamanca, Spain; 4 Department of Pathology, University Hospital of Salamanca, Salamanca, Spain; 5 Department of Pathology, University Hospital 12 de Octubre, Madrid, Spain; 6 Department of Neurosurgery, University Hospital of Salamanca, Salamanca, Spain; 7 Department of Oncology, University Hospital of Salamanca, Salamanca, Spain; 8 Department of Statistics, University of Salamanca, Salamanca, Spain; University of Navarra, Spain

## Abstract

Malignant astrocytomas are the most aggressive primary brain tumors with a poor prognosis despite optimal treatment. Dysfunction of mismatch repair (MMR) system accelerates the accumulation of mutations throughout the genome causing uncontrolled cell growth. The aim of this study was to characterize the MMR system defects that could be involved in malignant astrocytoma pathogenesis. We analyzed protein expression and promoter methylation of MLH1, MSH2 and MSH6 as well as microsatellite instability (MSI) and MMR gene mutations in a set of 96 low- and high-grade astrocytomas. Forty-one astrocytomas failed to express at least one MMR protein. Loss of MSH2 expression was more frequent in low-grade astrocytomas. Loss of MLH1 expression was associated with *MLH1* promoter hypermethylation and *MLH1* -93G>A promoter polymorphism. However, MSI was not related with MMR protein expression and only 5% of tumors were MSI-High. Furthermore, the incidence of tumors carrying germline mutations in MMR genes was low and only one glioblastoma was associated with Lynch syndrome. Interestingly, survival analysis identified that tumors lacking MSH6 expression presented longer overall survival in high-grade astrocytoma patients treated only with radiotherapy while MSH6 expression did not modify the prognosis of those patients treated with both radiotherapy and chemotherapy. Our findings suggest that MMR system alterations are a frequent event in malignant astrocytomas and might help to define a subgroup of patients with different outcome.

## Introduction

Malignant gliomas account for 70% of all primary brain tumors with an incidence rate adjusted to the European Standard Population of 5.27 per 100 000 persons per year [1]. Unfortunately, the majority of these patients display progressive disease and subsequent death. The most common and devastating brain tumor in adults is glioblastoma (grade IV) with a median survival of approximately 12-14 months despite optimal treatment [2,3]. Patients with anaplastic astrocytoma (grade III) survive for nearly 1.5 years, and those with low-grade astrocytomas (grade II) can survive for as long as 5-10 years [4,5]. Initiation and progression of malignant astrocytomas are related to their genetic and chromosomal alterations. In this context, recent molecular and genetic studies have identified different markers that help to determine prognosis and likelihood of therapeutic response [6-10].

Mismatch repair (MMR) system maintains DNA stability by repairing DNA mismatches and insertion/deletion loops acquired during DNA replication. Therefore, MMR system maintains genomic integrity and provides tumor suppressor functions. Defective MMR function is found both in sporadic tumors and in cancers related to Lynch syndrome [11] that is characterized by a predisposition to early onset tumors in the proximal colon as well as extracolonic malignancies such as astrocytomas [12-14]. This syndrome is due to germline mutations in one of the MMR genes, mostly *MSH2* or *MLH1*, and less frequently *MSH6* or *PMS2* [15,16]. Mutations in these genes result in microsatellite instability (MSI) and/or loss of expression of the associated protein. However, MMR deficiency in sporadic cancers is mostly due to loss of MLH1 expression as a result of somatic hypermethylation of its promoter [16]. *MLH1* promoter hypermethylation has been associated in colorectal cancer (CRC) with the *MLH1* -93G>A promoter polymorphism [17,18].

We have performed a molecular characterization of MMR system defects in malignant astrocytomas and we have evaluated the influence of these alterations in patient outcome. Specifically, we have investigated the expression profile and the promoter hypermethylation status of *MLH1*, *MSH2* and *MSH6* genes, as well as the MSI levels in pretreated low- and high-grade primary astrocytomas. We have also conducted a mutational analysis of MMR genes in tumors with MMR defective function.

## Materials and Methods

### Ethics Statement

The study was approved by the local Ethics Committees of the University Hospital of Salamanca (Salamanca, Spain) and University Hospital 12 de Octubre (Madrid, Spain), and written consent was obtained from the patients. The study was conducted according to the principles expressed in the Declaration of Helsinki.

### Patients and samples

A total of 96 newly diagnosed patients with primary astrocytoma grades II to IV (study cohort) were recruited from June 2000 until March 2006 at the University Hospital of Salamanca (Spain). Patients were followed up from diagnosis to the present at the Neurosurgery and Oncology Departments. Tumors were classified as 20 low-grade astrocytomas (grade II), 19 anaplastic astrocytomas (grade III) and 57 glioblastomas (grade IV) according to the 2007 WHO classification [19]. The clinicopathological features of the patients are summarized in Table 1 and Table S1.

**Table 1:** Summary of astrocytoma patient characteristics.

Patients, No. (%)	**LGA (n=20**)	**AA (n=19**)	**GBM (n=57**)
**Sex**			
Male	11 (55)	12 (63)	36 (63)
Female	9 (45)	7 (37)	21 (37)
**Median age, years [quartiles**]	35 [30.3-46.0]	57 [47.0-66.0]	63 [54.5-69.0]
**Tumor Region**			
Temporal	7 (35)	7 (37)	20 (35)
Frontal	6 (30)	5 (26)	22 (39)
Parietal	2 (10)	1 (5)	6 (10)
Occipital	0 (0)	2 (11)	5 (9)
Other	5 (25)	4 (21)	4 (7)
**Tumor Side**			
Right	8 (40)	10 (53)	34 (60)
Left	7 (35)	5 (26)	19 (33)
Other	5 (25)	4 (21)	4 (7)
**Surgery**			
Total resection	12 (60)	13 (69)	41 (72)
Subtotal resection	7 (35)	5 (26)	12 (21)
Partial resection	1 (5)	1 (5)	4 (7)
**Treatment**			
No treatment	10 (50)	4 (21)	5 (9)
Radiotherapy	5 (25)	8 (42)	39 (68)
Radiotherapy and Chemotherapy	5 (25)	7 (37)	13 (23)

LGA: low-grade astrocytoma, AA: anaplastic astrocytoma, GBM: glioblastoma.

An independent cohort of 71 newly diagnosed patients with primary astrocytomas WHO grades III and IV (12 anaplastic astrocytomas and 59 glioblastomas) [19] was explored to validate the prognostic results. Patients were admitted from April 2004 until December 2010 to the University Hospital 12 de Octubre (Madrid, Spain) and followed up from diagnosis to the present at the Neurosurgery and Oncology Departments. The clinical characteristics of this validation cohort are listed in Table S1.

All blood and tissue samples were obtained at diagnosis before initiation of treatment. Matched DNA from peripheral blood and frozen tumor specimens were extracted by standard phenol/chloroform procedure. Tumor tissues were also fixed in formalin and embedded in paraffin for immunohistochemical analysis.

### Tissue microarray design and Immunohistochemistry

**Figure 1:**
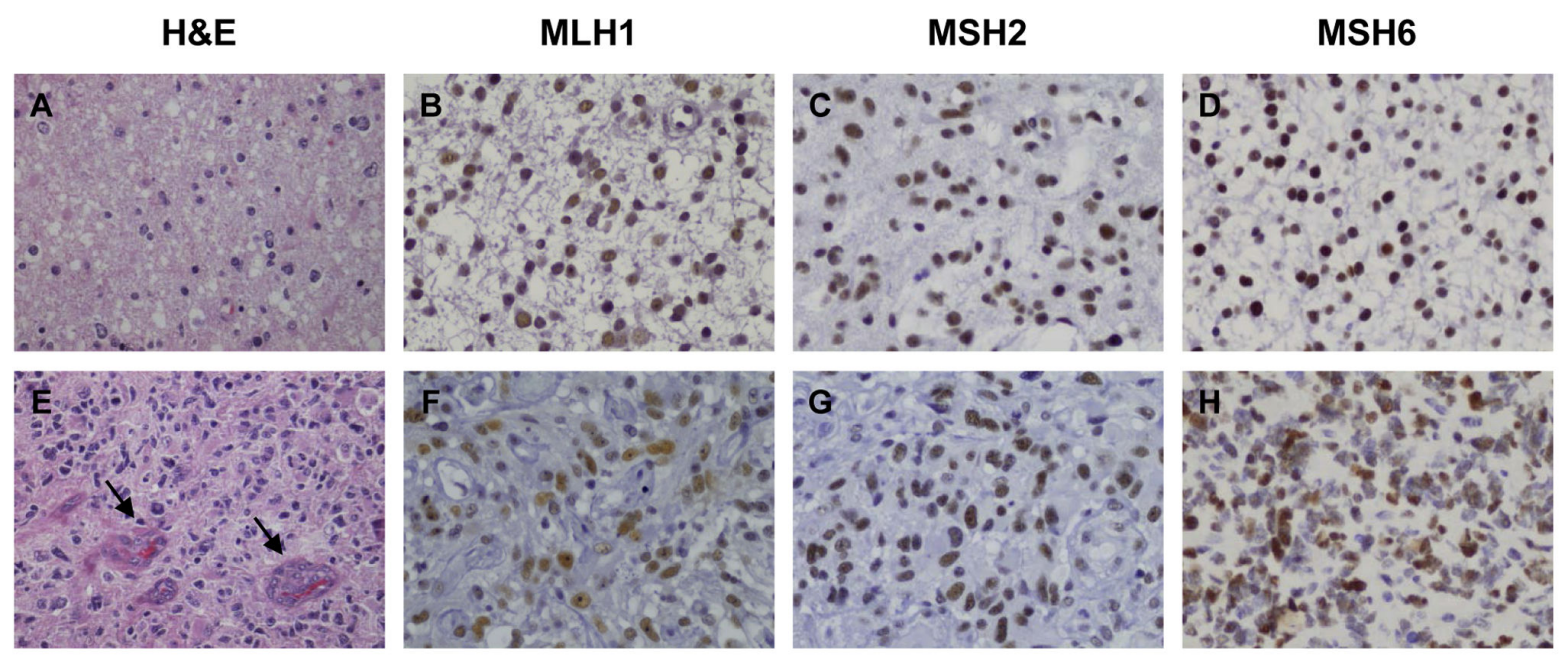
Representative images of hematoxylin and eosin (H&E), MLH1, MSH2 and MSH6 staining on paraffin-embedded sections of samples from representatives low-grade astrocytomas (grade II) (A-D) and glioblastomas (grade IV) (E-H) according to the 2007 WHO classification [19]. Low-grade astrocytomas are well differentiated and slow-growing tumors with absence of necrosis and microvascular proliferation, whereas high-grade astrocytomas are characterized by high cellularity and mitotic activity, necrosis and microvascular proliferation (arrows). MLH1, MSH2 and MSH6 expression was visualized by staining with specific antibodies and their expression was considered positive when nuclear staining was detected in more than 50% of tumor cells (Magnification x400).

Formalin-fixed paraffin-embedded tissue samples of all patients of the study cohort were used to prepare a tissue microarray (TMA) made with a tissue arrayer device (Beecher Instrument, MD). All astrocytomas were histologically reviewed and three 1-mm-diameter cylinders from different areas of morphologically representative non-necrotic sites of each tumor were included to ensure the quality, reproducibility and homogenous staining of the slides. Thus, 3 different TMA blocks were constructed, each containing the 96 astrocytomas and 8 different tissue controls (lymph node, skeletal muscle, cerebral cortex, cerebellum, spleen, adrenal gland, lung and kidney). Immunohistochemical staining was performed on these sections using MLH1 clone G168-15 (BD Pharmingen, San Diego, CA, USA), MSH2 clone FE11 (Biocare Medical, Concord, CA, USA) and MSH6 clone BC/44 (Biocare Medical, Concord, CA, USA) antibodies. MSH6 immunohistochemical staining was also performed in formalin-fixed, paraffin-embedded tumor tissue sections from the 72 patients in the validation cohort. MMR protein expression was evaluated semiquantitatively by a pathologist (A.S-B) and an author (I.R-H) blinded to clinical and molecular information and disagreements between reviewers were resolved by the analysis of the slides by a third author (R.G-S). Tumor area was considered positive when there was obvious nuclear staining in more than 50% of tumor cells. Staining of nuclei of adjacent normal cells and tumor-infiltrating lymphocytes was used as internal positive controls. Figure 1 shows representative examples of low- and high-grade astrocytomas staining.

### Methylation-specific multiplex ligation-dependent probe amplification (MS-MLPA)

The SALSA MS-MLPA Kit ME011 (MRC-Holland) was used to detect aberrant methylation in *MLH1*, *MSH2* and *MSH6* promoter regions using probes that recognized sequences containing a methylation-sensitive HhaI restriction site. The kit included 5 specific probes for *MLH1* (located at -659, -383, -246, -13 and +206 relative to initiating ATG), 3 probes for *MSH2* (located at -269, -193 and +124 to initiating ATG) and 3 probes for *MSH6* promoter region (located at -317, -126 and -32 to initiating ATG). All reactions were carried out as described by the manufacturer with minor modifications using in each reaction 150 ng of tumor DNA. PCR reaction fragments were separated by capillary gel electrophoresis (ABI Prism 3100 Genetic Analyzer, Applied Biosystems) and quantified using Genemapper software (Applied Biosystems). MS-MLPA processing was performed using Coffalyser analysis tool developed at MRC-Holland and tumor samples with a cut off value >0.75 was considered to be extensively hypermethylated as described by Jeuken et al [20]. For *MLH1* analysis, methylation status was calculated considering only the two specific probes related to gene silencing (-246 and -13 positions corresponding to C and D promoter regions) [21].

### Determination of MLH1 -93G>A genotype

Genotyping was performed using DNA extracted from peripheral blood of 96 patients and 200 sex-matched healthy subjects over 60 years old without history of cancer. *MLH1* -93G>A promoter polymorphism (rs1800734) status was determined using TaqMan SNP Genotyping Assay ID C_7535141 (Applied Biosystems) containing sequence-specific forward and reverse primers to amplify the polymorphic sequence and two probes labeled with VIC and FAM dyes to detect both alleles [22]. PCR reactions were carried out using TaqMan universal PCR Master Mix (Applied Biosystems) following manufacturer’s instructions in a Step-One Plus Real-time PCR system (Applied Biosystems). Genotype distribution in the control group was within the Hardy-Weinberg equilibrium (*P* > 0.1).

### Microsatellite Instability (MSI)

MSI was assessed by PCR in paired peripheral blood and tumor DNA obtained from 88 patients using a panel of 8 markers: 3 mononucleotide markers (BAT25, BAT26 and BAT40), 3 dinucleotide markers (D2S123, D5S346 and D17S250) and 2 tetranucleotide markers (MYCL and PAX6). This MSI marker set included the National Cancer Institute recommended markers for MSI detection in Lynch syndrome [11,23]. The 5’ antisense primers were labeled with FAM for BAT26, D5S346, D17S250 and D2S123, HEX for BAT40, MYCL and PAX6, and TET for BAT25. PCR reactions were performed using the Go Taq Hot Start Polymerase (Promega). PCR products were separated using an ABI Prism 3100 Genetic Analyzer (Applied Biosystems) and results were analyzed with Genemapper software (Applied Biosystems). Tumors were classified as MSI-High (MSI-H) if ≥ 30% markers demonstrated instability, MSI-Low (MSI-L) if < 30% demonstrated MSI, and microsatellite stable (MSS) if no marker exhibited MSI according to the established criteria for MSI determination [11,23].

### Mutational analysis of MLH1, MSH2 and MSH6 genes

Mutational analysis of MMR genes was performed by PCR-CSGE (Conformation-Sensitive Gel Electrophoresis) [24] in tumor DNA of patients with negative immunohistochemical staining for MLH1, MSH2 and/or MSH6 and in patients with MSI-H tumors. Those samples that showed a mobility shift in the CSGE analysis were additionally analyzed by direct sequencing to identify the nature of mutations. In addition, DNA extracted from peripheral blood was analyzed to determine the somatic or germline origin of tumor mutations. Primers sequences are available upon request.

### Statistical analysis

In most analysis, WHO grade III and IV astrocytomas are analyzed together as high-grade astrocytomas. The results were expressed as percentages for categorical variables and as medians [quartiles] for continuous variables. Associations between molecular and clinicopathological features as well as Hardy-Weinberg equilibrium were analyzed using the χ^2^ contingency test and the Fisher’s exact test when necessary (expected values below 5). Survival models were used to find clinical and/or molecular parameters related to overall survival (OS). OS was defined as the time between diagnosis and death or last follow-up. Those patients lost during follow-up were censored at the last known follow-up date and patients with a survival time lesser than 30 days were eliminated since these patients might have died for reasons other than the disease itself. Survival curves were estimated by the Kaplan Meier method and compared among patient subsets using the log-rank test. Multivariate Cox model was used to identify independent prognostic factors correlated with survival in astrocytoma patients. All categories were first ordered from good to bad with respect to prognosis. Then the corresponding Hazard Ratios (HR) will be greater than one helping the interpretation. Differences with a *P*-value <0.05 were considered as statistically significant and all tests were two-sided. All statistical analyses were performed using SPSS software.

## Results

### MMR protein expression

MLH1, MSH2 and MSH6 protein expression was determined in the 96 tumor tissues included in the tissue microarray. Analysis of MMR protein expression showed that 41 tumors (43%) presented loss of expression of at least one MMR protein. Loss of MLH1 expression was observed in 17 cases (18%), MSH2 in 21 cases (22%) and MSH6 in 31 cases (32%). In addition, 10 tumors failed to express two MMR proteins simultaneously (MLH1 and MSH2 expression were lost in 1 case, MLH1 and MSH6 expression in 4 cases, and MSH2 and MSH6 expression in 5 cases). Nine tumors did not express the three MMR proteins. Furthermore, MSH2 negative staining was significantly more frequent in low-grade astrocytomas (45%) than in high-grade astrocytomas (16%) (*P*=0.012), whereas MLH1 and MSH6 expression were not associated with tumor histopathology (Table 2).

**Table 2:** Relation between tumor grade, promoter methylation status and MLH1, MSH2 and MSH6 protein expression.

Patients, No. (%)	**MLH1 Expression**			**MSH2 Expression**				**MSH6 Expression**			
	**Positive**	**Negative**	***P*-value**		**Positive**	**Negative**	***P*-value**		**Positive**	**Negative**	***P*-value**
**Tumor grade**			0.721				**0.012**				0.805
Low-grade astrocytomas	17 (85)	3 (15)			11 (55)	9 (45)			14 (70)	6 (30)	
High-grade astrocytomas	62 (82)	14 (18)			64 (84)	12 (16)			51 (67)	25 (33)	
Promoter Methylation*			**0.021**				0.699				0.713
Hypermethylated	6 (54)	5 (46)			8 (73)	3 (27)			5 (62)	3 (38)	
No hypermethylated	70 (86)	11 (14)			64 (79)	17 (21)			57 (68)	27 (32)	

(* MLH1, MSH2 and MSH6 expression was associated with *MLH1*, *MSH2* and MSH6 promoter methylation status respectively)

### MMR promoter methylation

MMR promoter methylation was studied in 92 patients. Promoter hypermethylation of *MLH1* was present in 11 cases (12%), *MSH2* in 11 cases (12%) and *MSH6* in 8 cases (9%). No differences in MMR methylation levels were identified between different tumor grades. Methylation status of *MLH1* promoter was significantly associated with loss of MLH1 expression (*P*=0.021). Specifically, 46% of tumors with *MLH1* promoter hypermethylation showed lack of MLH1 expression (Table 2). We did not find any association between *MSH2* and *MSH6* promoter methylation and their protein expression (Table 2).

### Analysis of MLH1 -93G>A polymorphism

*MLH1* -93G>A polymorphism was determined in 96 astrocytoma patients and 200 control subjects. The distribution of genotypes in control samples did not significantly differ from that expected from Hardy-Weinberg equilibrium. *MLH1* -93GG genotype was found in 57 (60%), GA genotype in 31 (32%) and AA genotype in 8 (8%) patients. We did not find significant differences in distribution of *MLH1* -93G>A genotypes between low-grade astrocytomas, anaplastic astrocytomas and control subjects. However, carriers of the *MLH1* -93AA genotype were more represented in the group of glioblastoma patients (*P*=0.017) (Table 3). Furthermore, the AA genotype was more frequent in tumors with *MLH1* promoter hypermethylation (*P*<0.001) and in tumors that showed lack of MLH1 expression (*P*=0.030) (Table 3).

**Table 3:** Distribution of *MLH1* -93G>A genotypes according to diagnostic group, *MLH1* promoter hypermethylation and MLH1 protein expression.

Patients, No. (%)	**Genotype**		
	**GG + GA**	**AA**	***P*-value**
**Diagnostic group**			
LGA	19 (95)	1 (5)	0.540
AA	19 (100)	0 (0)	1.000
GBM	50 (88)	7 (12)	**0.017**
Controls	193 (96)	7 (4)	
***MLH1* Hypermethylation**			**< 0.001**
Hypermethylated	6 (55)	5 (45)	
No hypermethylated	78 (96)	3 (4)	
**MLH1 Expression**			**0.030**
Positive	75 (95)	4 (5)	
Negative	13 (76)	4 (24)	

### Microsatellite Instability

MSI analysis was performed in 88 cases and revealed that the most frequent unstable markers in our series were BAT25 (15% of cases) and BAT40 (13% of cases). On the contrary, BAT26 was unstable in 1% of cases.

Forty-seven patients (53%) were classified as MSS, 37 patients (42%) as MSI-L and 4 patients (5%) as MSI-H, with no differences between tumor grades. The four MSI-H tumors were glioblastomas. Three of these cases showed expression of all MMR proteins and one case presented loss of MSH6 expression. Neither MMR protein expression nor MMR promoter methylation nor *MLH1* -93AA genotype were associated with MSI status (Table S2).

### Germline and somatic mutations of MMR genes

Mutation analysis of MMR genes was performed in the forty-four tumors that showed abnormalities in MMR protein expression or MSI-H. Six of these tumors carried mutation in *MLH1*, *MSH2* or *MSH6* genes (Table 4). Four tumors presented MSS with MMR germline mutations and lack of expression of the associated protein; one glioblastoma with MSI-L showed two mutations in the *MSH2* gene, one mutation in the *MLH1* gene and loss of MLH1, MSH2 and MSH6 protein expression. The remained case was a MSI-H glioblastoma with loss of MSH6 expression and a family history of colorectal cancer. This patient showed a pathogenic germline frameshift mutation in *MSH2* gene and wild-type allele loss in tumor tissue.

**Table 4:** MMR gene mutations identified in tumors with loss of at least one MMR protein expression and/or MSI-H.

**MSI**	**Case**	**Gene**	**MMR Germline Mutation**	**MMR Somatic Mutation**	**Pathogenicity**	**Loss of MMR expression**
MSS	GBM	*MLH1*	c.2146GA (p.Val716Met)[25]		Uncertain	MLH1, MSH2
MSS	LGA	*MSH2*	c.1159CG (p.Leu387Val)		Uncertain	MSH2
MSS	LGA	*MSH6*	c.4004AC (p.Glu1335Ala)		Uncertain	MLH1, MSH2, MSH6
MSS	GBM	*MSH6*	c. *(24_28)delGTTGA		Uncertain	MSH6
MSI-L	GBM	*MLH1*		c.1937AG (p.Tyr646Cys)[26]	Uncertain	MLH1, MSH2, MSH6
		*MSH2*		c.1983delA (p.Lys661AsnfsX24)	Pathogenic	
		*MSH2*		c.1064GA (p.Arg355Lys)	Uncertain	
MSI-H	GBM	*MSH2*	c.2239_2240delAT (p.Ile747ArgfsX2)[27]	Wt allele loss in tumor	Pathogenic	MSH6

We described for the first time in this study three novel mutations in *MSH2* gene c.1159CG (p.Leu387Val), c.1983delA (p.Lys661AsnfsX24) and c.1064GA (p.Arg355Lys), and two novel mutations in *MSH6* gene c.4004AC (p.Glu1335Ala) and c. *(24_28) delGTTGA (Table 4).

### Impact of MMR alterations on patient survival

Next, we investigated the prognostic impact of both MMR alterations and clinical parameters on 92 astrocytoma patients (20 low-grade astrocytomas and 72 high-grade astrocytomas). Univariate survival analysis revealed that loss of MSH6 expression was significantly associated with a better median OS in high-grade astrocytomas (13.8 months vs. 10.1 months) (Table 5A). Age of patients (<60 years vs. ≥60 years) and treatment (radiotherapy and chemotherapy vs. radiotherapy alone vs. no treatment) were also significantly associated with OS in high-grade astrocytomas (Table 5A). Furthermore, multivariate Cox model indicated that MSH6 expression, age and treatment were statistically significant independent prognostic factors for OS of high grade-astrocytomas in our series (Table 5A).

**Table 5:** Univariate and multivariate survival analysis in high-grade astrocytomas.

**Parameter**	**Univariate Analysis**		**Multivariate Analysis**	
	**HR (95% C.I.**)	***P*-value**	**HR (95% C.I.**)	***P*-value**
**A**)** STUDY COHORT (n=72**)				
MSH6 expression	1.76 (1.01-3.07)	**0.045**	1.84 (1.05-3.23)	**0.033**
Age	2.03 (1.18-3.50)	**0.009**	1.75 (1.02-3.02)	**0.042**
Treatment	2.53 (1.56-4.11)	**0.001**	2.42 (1.52-3.87)	**< 0.001**
**B**)** VALIDATION COHORT (n=71**)				
MSH6 expression	1.30 (0.66-2.56)	0.443	1.56 (0.77-3.19)	0.219
Age	1.76 (0.99-3.12)	0.051	1.74 (0.98-3.11)	0.060
Treatment	1.55 (1.01-2.47)	**< 0.001**	1.70 (1.02-2.84)	**0.041**

In order to validate the prognostic value of MSH6 expression on high-grade astrocytomas survival, we evaluated MSH6 protein expression in an independent cohort of 71 high-grade astrocytomas. Fifteen (21%) of these tumors showed loss of MSH6 expression (Figure S1). However, survival analysis revealed that MSH6 expression was not associated with prognosis, whereas treatment was an independent prognostic factor for OS and there was marginally significant correlation between age and OS time in this validation group of patients (Table 5B).

**Figure 2:**
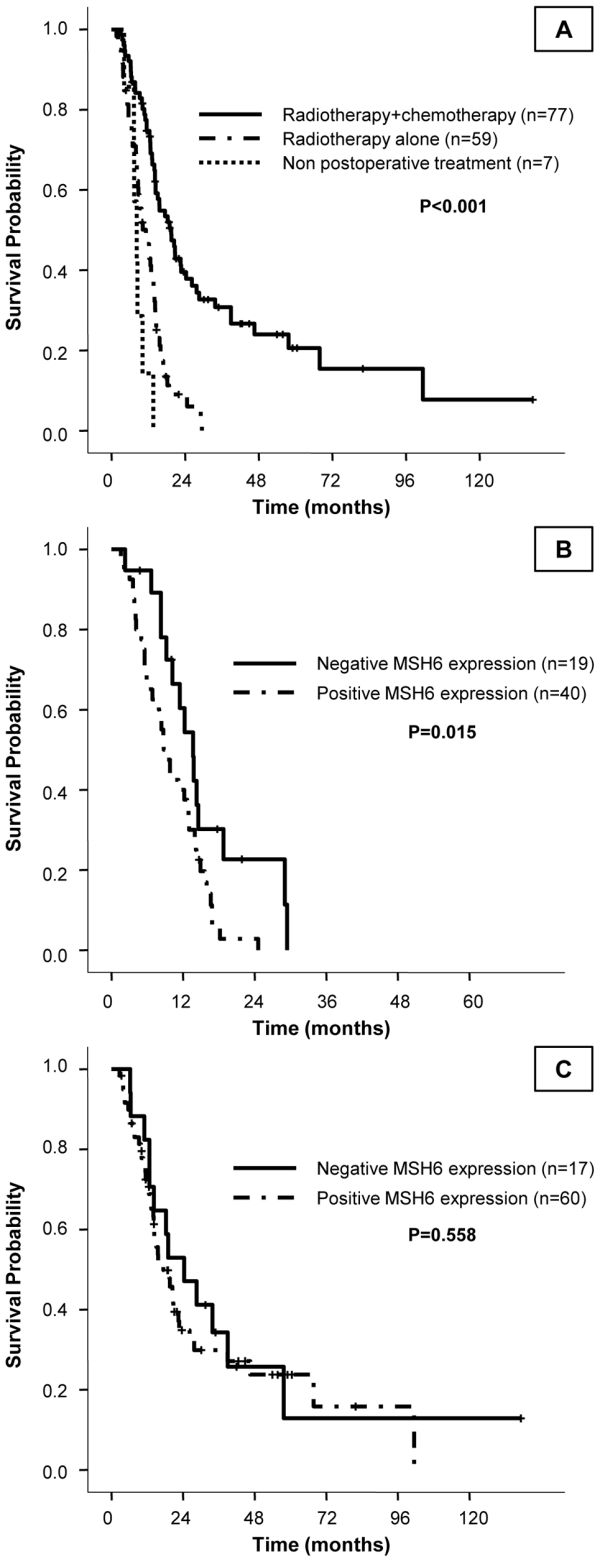
Kaplan-Meier estimates of overall survival in high-grade astrocytomas in the entire patient set according to treatment received. Treatment with both radiotherapy and chemotherapy confers a significant increase in overall survival time (A). Survival analysis in each treatment group separately showed that loss of MSH6 expression correlated with a better overall survival in patients receiving radiation therapy alone (B), whereas MSH6 expression did not modify prognosis of patients receiving both radiotherapy and chemotherapy (C).

We further analyzed the differences between the study cohort and the validation cohort of patients in order to unravel the role of MSH6 protein expression in patients diagnosed of astrocytoma. We found that the number of patients who received radiotherapy and chemotherapy was significantly higher in the validation cohort compared to the study cohort (Table 6). Consequently, those patients belonged to the validation cohort had a better outcome compared to the study cohort (Table 6). Since treatment itself constitute a strong prognostic marker (Figure 2A), we stratified the entire patient set by treatment and we analyzed the association between MSH6 expression and clinical outcome in each treatment subgroup separately. Loss of MSH6 expression was associated with a better median OS time in the group of high-grade astrocytoma patients only treated with radiotherapy (HR=2.17, 95% C.I. 1.14-4.11, *P*=0.015) (Figure 2B); meanwhile no significant differences were found in the group of patients treated with radiotherapy plus chemotherapy (HR=1.21, 95% C.I. 0.64-2.26, *P*=0.558) (Figure 2C). Survival analysis could not be estimated in the group of patients that did not receive any treatment due to small number of events.

**Table 6:** Differences in prognostic characteristics between the study cohort and the validation cohort of high-grade astrocytoma patients.

Patients, No. (%)	**Study cohort (n=72**)	**Validation cohort (n=71**)	***P*-value**
**MSH6 expression**			0.198
Positive	50 (69)	56 (79)	
Negative	22 (31)	15 (21)	
**Age**			0.359
<60	31 (43)	36 (51)	
≥60	41 (57)	35 (49)	
**Treatment**			**< 0.001**
No treatment	5 (7)	2 (3)	
Radiotherapy	47 (65)	12 (17)	
Radiotherapy and Chemotherapy	20 (28)	57 (80)	
**Median survival**, months [quartiles]	12.7 [10.1-15.3]	15.1 [10.8-19.4]	**0.012**

## Discussion

Malignant astrocytomas are one of the most devastating cancers with a dismal prognosis. Virtually all high-grade astrocytomas progress and locally relapse regardless of improved diagnosis and multi-modality treatment approach [2,3]. Therefore, identification of new markers may contribute to a better prediction of prognosis and response to therapy. Astrocytomas are characterized by an infiltrating and aggressive behavior directly related to their genetic alterations in core signaling pathways [28]. In this regard, MMR activity could be implicated in astrocytoma pathogenesis due to the fact that loss of MMR function accelerates the accumulation of mutations that are no longer repaired.

We have identified a large number of astrocytomas with defective MMR system expression. Forty-three percent of tumors included in our series failed to express at least one MMR protein at diagnosis, suggesting that this abnormality could be an intrinsic property of a subgroup of tumors. We also showed that MLH1 and MSH6 expression profiles were similar in both low- and high-grade astrocytomas whereas the lack of MSH2 expression was significantly more frequent in low-grade astrocytomas. In this sense, it has been previously reported an increased expression of MSH2 in high-grade astrocytomas compared with low-grade astrocytomas. Therefore, up-regulation of MSH2 levels may be related to an increased cell proliferation rate in astrocytomas [29,30]. Although MSH2 expression is typically lost in colorectal or endometrial tumors [13,14,16], high levels of MSH2 expression have also been described in more malignant and proliferative melanoma and salivary gland grade tumors [31,32].

Loss of protein expression could be due to aberrant DNA methylation of cytosine residues in CpG promoter islands that leads to transcriptional silencing of the associated genes [33]. We have found a specific association between *MLH1* methylation of the proximal promoter region and the absence of MLH1 expression in astrocytomas. Thus, we further confirm that *MLH1* proximal promoter methylation is important in inhibiting *MLH1* transcription as it has been previously reported in colorectal cancer (CRC) [21]. Moreover, our results suggest that MSH2 and MSH6 expression are not regulated by their promoter methylation status in astrocytomas.

Analysis of the genotypic distribution of *MLH1* -93G>A polymorphism, located in a promoter region required for maximal transcriptional activity [34], showed that the -93AA genotype was associated with *MLH1* promoter methylation and deficient MLH1 expression never reported before in astrocytomas. The -93AA genotype was also associated with a higher risk of developing glioblastomas. This variant has been previously associated with loss of functional MMR system in colorectal and endometrial cancers [17,18,35] and with an increased risk of developing different tumors [17,36,37]. Nevertheless, further studies in larger series of patients are necessary to confirm our observation.

MSI analysis revealed a low incidence of MSI-H tumors in our series (5%) similar to that previously reported [38-41]. All MSI-H cases were classified as glioblastomas, suggesting a possible relation with higher proliferation cell levels. MSI is a molecular feature resulting mainly from inactivating alterations of the MMR system; however, loss of MMR protein expression was not related to MSI status in our study. MMR deficiencies are well associated with MSI status in several tumors, such as ovarian, endometrial or colorectal cancers [11,14,42], but in other tumors such as medulloblastoma or Ewing sarcoma this association has not been reported [43,44]. These findings suggest that MMR protein deficiencies are related to MSI depending on the tumor type [44]. In addition, we observed that BAT25 and BAT40 were the most frequent unstable markers in astrocytomas instead of BAT26 that is the most unstable marker in CRC. This difference might indicate that MSI in astrocytomas is promoted by different mechanisms than in CRC.

Search for mutations in MMR genes showed only two pathogenic mutations. A germline pathogenic mutation in *MSH2* gene [27] was found in a MSI-H glioblastoma belonged to a Lynch syndrome family. The other pathogenic mutation was a novel somatic *MSH2* mutation in a MSI-L glioblastoma that also carried two additional variants of unknown significance in *MSH2* and *MLH1* genes. One *MLH1*, one *MSH2* and two *MSH6* mutations of unknown significance that could be causing the lack of expression of the associated proteins were also detected. The absence of MMR gene mutations in most of the sporadic MSI-H astrocytomas was in accordance with data reported from sporadic CRC with high level of MSI that do not harbor mutations in the repair genes [15,16].

Loss of MSH6 expression was more common than the absence of MLH1 or MSH2 expression in our series, suggesting an important role of this protein in the pathogenesis of astrocytomas. It has been recently reported that MSH6 alterations arise in gliomas as a consequence of temozolomide treatment [45,46]. Nevertheless, MSH6 alterations have also been documented in pretreated astrocytoma tumors [47,48]. We must note that all cases included in our study were analyzed before therapy, confirming that loss of MSH6 expression in astrocytomas is not always secondary to therapy-induced mutagenesis.

Furthermore, survival analysis showed that loss of MSH6 expression was significantly associated with longer overall survival in high-grade astrocytoma patients in our series. Controversially, we did not find this association when we analyzed MSH6 expression in an independent validation cohort of high-grade astrocytoma patients. However, the treatment approach was different between the two cohorts of patients due to the different recruitment period of patients in each series. For decades, postoperative radiotherapy has been the standard treatment for newly diagnosed high-grade astrocytomas [49]. However, since the 2005 publication of Stupp regimen [50], the established standard therapy for newly diagnosed high-grade astrocytomas has been surgical resection followed by radiotherapy with concomitant and adjuvant chemotherapy with temozolomide with the consequent significant increase in overall survival time [3,50,51]. Therefore, survival analysis was performed in each treatment group separately and revealed that loss of MSH6 expression was significantly associated with longer overall survival in patients with high-grade astrocytomas treated with radiotherapy alone, whereas no differences were found in those patients that received radiotherapy plus chemotherapy. The chemotherapy agent used in the majority of these patients was the alkylating agent temozolomide.

Several studies have suggested that loss of MSH6 activity confers resistance to temozolomide treatment in glioma and may therefore contribute to progressive tumor growth and tumor recurrence [45,46]. However, several analyses concluded that MMR deficiency does not play a role in clinical resistance to alkylator therapy in malignant gliomas [47]. This finding was in accordance to our results that MSH6 expression does not modify the prognosis of those patients treated with radiotherapy and chemotherapy. On the other hand, the roles of MMR deficiency in radiotherapy response are less clear. In our series, loss of MSH6 expression confers a better prognosis in high-grade astrocytomas treated only with radiotherapy suggesting that MSH6 protein could modulate response to radiation therapy in these tumors. It has been reported that loss MSH6 expression in initial lesions was indicator of prolonged survival in a group of patients treated mostly with postoperative radiotherapy [48]. Ionizing radiation (IR) exposure induces a wide variety of lethal DNA damage, especially double-strand breaks (DSB). Although MSH6 plays a fundamental role in the repair of mismatched DNA bases, recent studies demonstrated that MSH6 contributes to DSB repair though the non-homologous end-joining (NHEJ) pathway following IR exposure by the interaction of MSH6 with Ku70 [52]. Thus, MSH6-deficient cells have a major DSB repair defect and are more sensitive to IR-induced cell death [52]. Therefore, our results indicate that MSH6 expression might constitute a prognostic marker for astrocytoma survival in patients treated only with radiotherapy.

In summary, our results demonstrate that MMR system alterations are a frequent event in malignant astrocytomas. We suggest that analysis of MMR genes allows to define a subset of astrocytomas with different outcome and could help to search for new therapeutic strategies.

## Supporting Information

Table S1 **Detailed clinical characteristics of the study and validation cohort of astrocytoma patients.**
(XLS)

Table S2 **MLH1, MSH2 and MSH6 expression and methylation status according to MSI levels.**
(DOC)

Figure S1 **Representative examples of negative (A, B) and positive (C, D) MSH6 staining in four glioblastoma tumors (WHO grade IV).** MSH6 expression was analyzed in formalin fixed, paraffin embedded tumor sections from all patients in the study cohort and the validation cohort. MSH6 staining on these sections was performed using MSH6 clone BC/44 (Biocare Medical, Concord, CA, USA) antibody and counterstained with hematoxylin and eosin. MSH6 staining was considered positive when there was obvious nuclear staining in more than 50% of tumor cells (Magnification, x400).
(TIF)
