# Supplementary material for: Integrated Analysis of Mismatch Repair System in Malignant Astrocytomas
Source: PLoS One. 2013 Sep 20;8(9):e76401. doi: 10.1371/journal.pone.0076401 (PMC3779191; doi:10.1371/journal.pone.0076401)
Supplement: Table S2 — MLH1, MSH2 and MSH6 expression and methylation status according to MSI levels. (DOC) [file pone.0076401.s002.doc]

**Table S2. MLH1, MSH2 and MSH6 expression and methylation status according to MSI levels.**

| Patients, No. (%) | **MSS** | **MSI-L** | **MSI-H** | ***P*-value** |
| --- | --- | --- | --- | --- |
| **MLH1 Expression** |  |  |  | 0.542 |
| Negative | 8 (17) | 8 (22) | 0 (0) |  |
| Positive | 39 (83) | 29 (78) | 4 (100) |  |
| ***MLH1* Methylation** |  |  |  | 0.543 |
| Hypermethylated | 6 (13) | 3 (8) | 1 (25) |  |
| No hypermethylated | 41 (87) | 34 (92) | 3 (75) |  |
| **MSH2 Expression** |  |  |  | 0.551 |
| Negative | 11 (23) | 8 (22) | 0 (0) |  |
| Positive | 36 (77) | 29 (78) | 4 (100) |  |
| ***MSH2* Methylation** |  |  |  | 0.525 |
| Hypermethylated | 4 (9) | 5 (14) | 1 (25) |  |
| No hypermethylated | 43 (91) | 32 (86) | 3 (75) |  |
| **MSH6 Expression** |  |  |  | 0.326 |
| Negative | 12 (26) | 15 (41) | 1 (25) |  |
| Positive | 35 (74) | 22 (59) | 3 (75) |  |
| ***MSH6* Methylation** |  |  |  | 0.135 |
| Hypermethylated | 2 (4) | 6 (16) | 0 (0) |  |
| No hypermethylated | 45 (96) | 31 (84) | 4 (100) |  |
| **-93G>A *MLH1* polymorphism** |  |  |  | 0.525 |
| GG+GA genotype | 43 (91) | 34 (92) | 3 (75) |  |
| AA genotype | 4 (9) | 3 (8) | 1 (25) |  |
