# Supplementary figures and images for: Integrated Analysis of Mismatch Repair System in Malignant Astrocytomas
Source: PLoS One. 2013 Sep 20;8(9):e76401. doi: 10.1371/journal.pone.0076401 (PMC3779191; doi:10.1371/journal.pone.0076401)

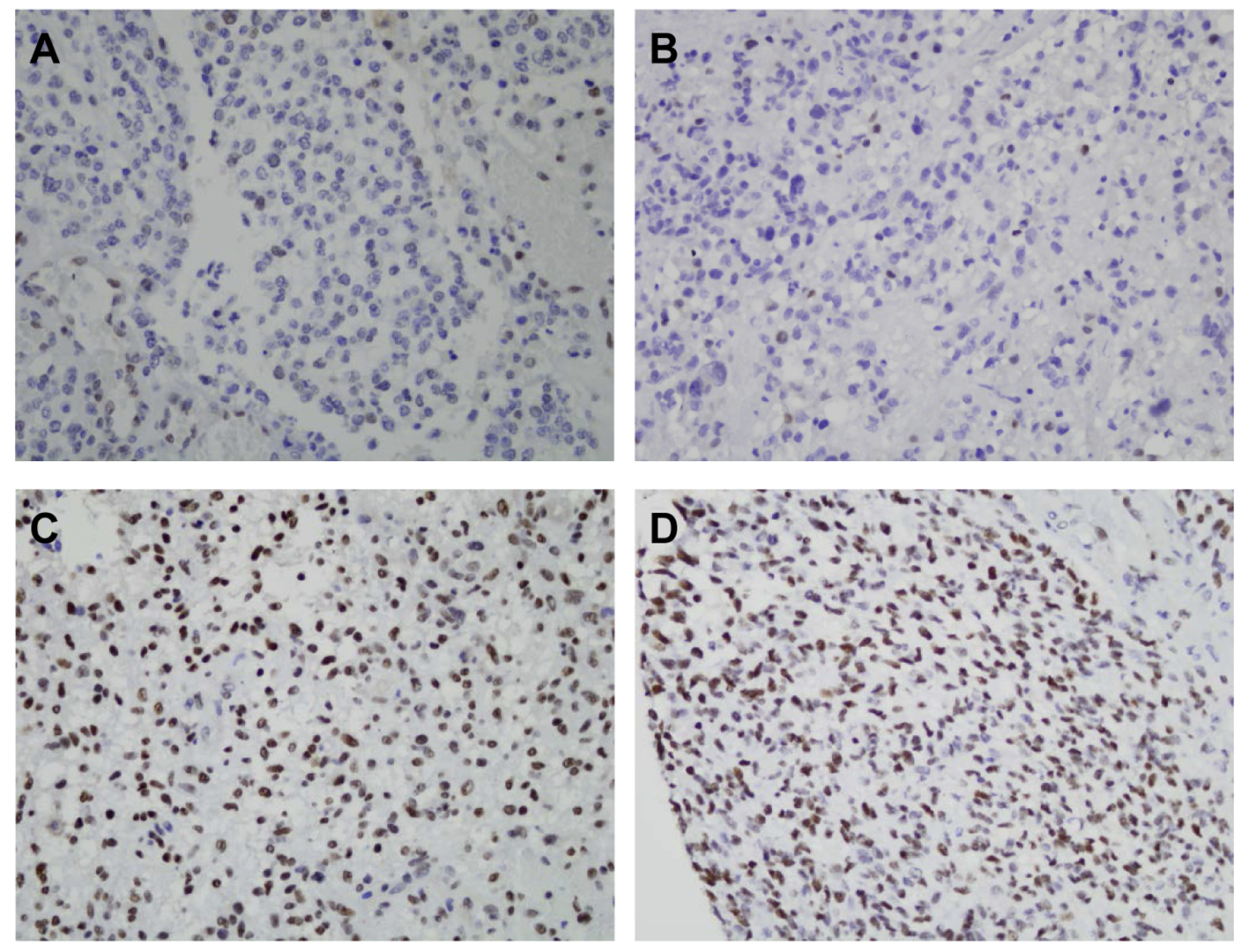

Supplement: Figure S1 — Representative examples of negative (A, B) and positive (C, D) MSH6 staining in four glioblastoma tumors (WHO grade IV). MSH6 expression was analyzed in formalin fixed, paraffin embedded tumor sections from all patients in the study cohort and the validation cohort. MSH6 staining on these sections was performed using MSH6 clone BC/44 (Biocare Medical, Concord, CA, USA) antibody and counterstained with hematoxylin and eosin. MSH6 staining was considered positive when there was obvious nuclear staining in more than 50% of tumor cells (Magnification, x400). (TIF) [file pone.0076401.s003.tif]
